# The Glass Half Empty: How Emotional Exhaustion Affects the State-Trait Discrepancy in Self-Reports of Teaching Emotions

**DOI:** 10.1371/journal.pone.0137441

**Published:** 2015-09-14

**Authors:** Thomas Goetz, Eva S. Becker, Madeleine Bieg, Melanie M. Keller, Anne C. Frenzel, Nathan C. Hall

**Affiliations:** 1 Department of Empirical Educational Research, University of Konstanz, Konstanz, Germany; 2 Thurgau University of Teacher Education, Kreuzlingen, Switzerland; 3 Department of Psychology, University of Munich, Munich, Germany; 4 Department of Educational and Counselling Psychology, McGill University, Montreal, Canada; Queen Mary University of London, UNITED KINGDOM

## Abstract

Following from previous research on intensity bias and the accessibility model of emotional self-report, the present study examined the role of emotional exhaustion in explaining the discrepancy in teachers’ reports of their trait (habitual) versus state (momentary, “real”) emotions. Trait reports (habitual emotions, exhaustion) were assessed via trait questionnaires, and state reports (momentary emotions) were assessed in real time via the experience sampling method by using personal digital assistants (*N* = 69 high school teachers; 1,089 measures within teachers). In line with our assumptions, multi-level analyses showed that, as compared to the state assessment, teachers reported higher levels of habitual teaching-related emotions of anger, anxiety, shame, boredom, enjoyment, and pride. Additionally, the state-trait discrepancy in self-reports of negative emotions was accounted for by teachers’ emotional exhaustion, with high exhaustion levels corresponding with a greater state-trait discrepancy. Exhaustion levels did not moderate the state-trait discrepancy in positive emotions indicating that perceived emotional exhaustion may reflect identity-related cognitions specific to the negative belief system. Implications for research and educational practice are discussed.

## Introduction

### Self-reports of Trait versus Real-time Emotions in Teachers

The classroom environment represents a highly interactive and emotionally charged setting characterized by achievement striving, goal attainment, learning challenges, and interpersonal conflict [1]. Accordingly, students’ emotions in educational contexts have attracted increasing attention over the past 20 years with cumulative empirical evidence showing these emotions to influence students’ self-regulated behavior and motivational orientations, decision-making (e.g., to persist or drop out), and psychological well-being [2]. Although it can be assumed that emotions show those effects not only for students but also for teachers, there exists a surprising and unfortunate gap in educational psychology research on emotions of teachers [3].

With regard to research examining teachers’ emotions, the vast majority of studies to date have employed qualitative interviews (see [3]). However, as the samples for most qualitative studies are small, their contribution to our understanding concerning the typical intensity of specific teacher emotions is limited. Although quantitative approaches could provide more generalizable insight into the frequency and intensity of discrete teacher emotions, very few such studies exist. Of the existing quantitative studies conducted, most have employed state (momentary) and/or trait (habitual) assessments of teachers’ emotions with the results of these studies indicating higher levels of trait as compared to state reports.

One possible reason for such higher levels in trait reports might be peak effects, that is, the overweighting of very intense experiences in retrospective judgments [4, 5]. According to Robinson and Clore’s accessibility model of emotional self-report [6], it can further be concluded that individuals’ levels of emotional exhaustion should contribute to the state-trait discrepancy as they should strongly impact reports of trait, but not state, emotions. As emotional exhaustion represents a critical component of the negative belief system [7, 8] it can be assumed that it should impact specifically reports of negative trait emotions.

Based on those considerations, we focused in the present study on the following hypotheses: Teachers were assumed to report higher levels of both negative and positive trait teaching emotions as compared to their reports of state emotions. Further, teachers reporting greater emotional exhaustion were hypothesized to show a greater state-trait discrepancy in reports of negative emotions.

#### Findings on the Intensity of Teaching Emotions

There exist notably few quantitative studies investigating the intensity of the emotions experienced by teachers, with most research having been conducted recently utilizing both state and trait assessment methods. Thus, in contrast to research on students’ emotions in which trait assessments have almost exclusively been employed [2], quantitative findings concerning both reports of habitual and momentary emotions in teachers are available. Of the three studies in which both state and trait teaching emotions were assessed, two were diary studies [9, 10] focusing on anger, anxiety, and enjoyment, and the third was an experience sampling study [11] focusing on teachers’ anger, anxiety, shame, boredom, enjoyment, and pride. When contrasting the levels of state versus trait emotions (see state/trait comparisons with students; e.g., [12, 13]), all three studies showed higher levels on the trait assessment as compared to the state assessment.

#### Emotional Exhaustion as a Moderator of State-Trait Differences in Teaching Emotions

Why would teachers be expected to report higher levels of their trait as compared to state emotions? One potential reason involve “peak effects” that can be assumed to be common across individuals as opposed to person-specific in nature (e.g., [5, 14]. A second reason is based on the assumption that there exists a clear qualitative difference between state and trait reports of emotions. More specifically, trait emotions can be assumed to be more strongly influenced by semantic knowledge than state emotions. Based on previous findings on the discrepancy between trait and state emotional assessments, Robinson and Clore [6] propose an accessibility model of emotional self-report in which they distinguish between trait and state emotional self-reports, classifying them according to their respective memory systems. While trait emotions are semantic, conceptual, and decontextualized, state emotions are episodic, experiential, and contextual. Whereas state emotions are assumed to be directly assessed and, consequently, influenced by situational cues, reports on trait emotions are assumed to be strongly influenced by individuals’ beliefs and semantic knowledge (i.e., knowledge of which emotions should be experienced in specific situations) [4]. Consequently, a discrepancy between trait and state emotional assessments can be assumed with trait assessments relating more strongly to subjective beliefs, mainly identity-related beliefs, than state assessments. Such beliefs can be assumed to differ across persons and thus might account for interindividual variance in the state-trait discrepancy.

In more detail, one’s identity-related beliefs can pertain to beliefs about one’s emotions in general [6]. Accordingly, one’s perceived emotional exhaustion can represent a particularly salient identity-related belief, with exhaustion comprising the emotion-specific component of burnout in reflecting the belief that one has nearly depleted one’s emotional resources (e.g., [15]; sample scale items: “I feel emotionally drained from my work”, “I feel emotionally exhausted”).

Judgments of emotional exhaustion can be hypothesized as belonging to the negative belief system (see also [7]) and may therefore impact other beliefs within this system, such as reports of negative trait emotions. It is important to note that although emotional exhaustion can also be assumed to impact negative state emotions, this impact should be less strong than on trait negative emotions due to state emotions being less impacted by the belief system. Thus, emotional exhaustion can be assumed to be a moderator of the state-trait discrepancy for negative teaching emotions such that high levels of emotional exhaustion should correspond with greater state-trait discrepancies. Whether emotional exhaustion is also a moderator with respect to positive emotions remains an exploratory research question.

### The Present Research

We evaluated the hypothesis that teachers would report higher levels of both positive and negative emotions on trait-oriented, self-report measures as compared to state self-reports [4, 12, 16]. Further, we assumed that teachers with high levels of emotional exhaustion should show a greater state-trait discrepancy, specifically with respect to reports of their negative trait emotions, than teachers reporting low exhaustion levels [6].

The present study was conducted with teachers and assessed both teaching-related trait measures of emotions (anger, anxiety, shame, boredom, enjoyment, pride) as well as emotional exhaustion. Additionally, state self-reports of emotions were administered during regular classes. To evaluate the main study hypotheses, we adopted a multilevel modeling approach to account for the nested data structure (measures within persons). Given previous findings on possible gender differences in trait reports of academic emotions (e.g., [17]), gender was controlled in our analyses.

## Method

### Ethical Statement

The present study was conducted in compliance with ethical standards expressed in the WMA Declaration of Helsinki. The study has been approved and all study procedures have been deemed appropriate by the Institutional Review Board of the University of Konstanz (Reference No. IRB15KN008). Prior to participation, teachers were informed about the goals of the research, duration, procedure and anonymity of their data. Participation was voluntarily and it was possible to withdraw participation at any time. Verbal informed consent prior to data collection was provided by all teachers. This procedure was in compliance with ethical standards provided by the Federation of German Psychologists Association and the American Psychological Association. Guidelines provided by these institutions state that formal informed consent is not obligatory when no potential harm or distress is to be expected and/or when normal educational practices are followed as a goal of the research. Furthermore, all identifiers that could link individual participants to their results were removed and destroyed after data entry. Hence, analyses were conducted on anonymous data.

### Sample and data collection

The sample consisted of *N* = 69 teachers (30 female, 39 male; *M*_age_ = 42.69 years, *SD* = 10.30, range: 27–63) from 10 German secondary schools (Gymnasium, the highest track of the German school system with approximately one third of the total student cohort and an overall average of 58% female teachers) [18]. Participants had been teaching on average for 14.22 years (*SD* = 10.67). To ensure heterogeneous sampling with regard to subject domains of instruction, teachers were recruited across 27 different school subjects (e.g., mathematics, history, geography, English, French) with most of the participants teaching two or three subjects.

Trait and demographic data were assessed using a standardized paper-and-pencil questionnaire at the beginning of the study, after which state self-report measures were administered over the course of two weeks via the experience sampling method ([19, 20]; for adapting experiense-sampling protocols for teachers see [21]). State emotions were assessed via a digital questionnaire presented on a personal digital assistant (PDA). Following an instructional session by trained testing personal on the use of PDAs, teachers activated the PDA at the beginning of each regular lesson with each individual signal subsequently occurring at randomized intervals over the next 40 mins (altogether 1089 state assessments; *M* = 15.78 assessments per teacher, on average (*SD* = 10.01); range of assessment within teachers: 2–41). Upon hearing the signal, participants temporarily paused their instructional activities and completed the state questionnaire. It should be noted that teachers were informed to not interrupt instruction mid-sentence so as to minimize lesson intrusion, and to allow themselves five minutes to answer the questions upon hearing the signal. Concerning missing data, responses were not obtained after the five minute response period for 7.5% of the signals (most likely due to not having heard the auditory alert). Of the teachers who responded to questions on the PDA, the response rate was nearly 100% (only three missing responses on altogether 6,534 questions resulting from 6 items assessed in 1,089 lessons). Teachers required, on average, 38 seconds (*SD* = 26) to complete the state questionnaire; a notably brief period of interruption deemed acceptable by both school administrators and participating teachers.

### Measures

#### Teaching Emotions–State

In line with principles of ESM methodology (e.g., [13, 20]), state emotions were assessed using single items. The main reason for the use of single-item measures was their relatively greater validity as compared to longer multi-item state measures. In contrast to single item measures, multi-item measures require more time to respond thereby potentially assessing participants’ emotional responses to questionnaire completion rather than the teaching activity in which they are currently engaged.

In line with previous single-item assessments of emotions (e.g., [12, 13]), items were formulated as follows: “*How much* [EMOTION] *are you experiencing in this moment*?” with the emotions assessed including anger, anxiety, shame, boredom, enjoyment, and pride. The response format was a five-point Likert scale ranging from (1) “*not at all*” to (5) “*very strongly*.” Item means and standard deviations are shown in Table 1 with the intercorrelations outlined in Table 2. We calculated the intraclass correlation ICC(1) values for each state emotion to examine the proportion of variance in emotional experiences lying within teachers as compared to the total variance. Further, we calculated the ICC(2) values that are a function of both the ICC(1) and the average number of observations within participants (*n* = 15.78 in the present study). The ICC(2) can be interpreted in our study as a measure of the reliability of aggregated (i.e., mean) state scores [22]. The ICC(1) and ICC(2) values are outlined in Table 3. The ICC(1) values ranged from .200 to .435, implying that the largest proportion of variance of emotional experiences lay within teachers; the ICC(2) values ranged from .798 to .924 indicating a high degree of reliability in our aggregated measures.

**Table 1:** Descriptive Statistics and Mean Level Differences.

Measures	Total sample		Females		Males	
	*M*	*SD*	*M*	*SD*	*M*	*SD*
**State constructs–single items**						
Anger	1.61	0.49	1.61	0.44	1.61	0.53
Anxiety	1.08	0.17	1.08	0.17	1.09	0.17
Shame	1.09	0.20	1.09	0.20	1.08	0.21
Boredom	1.43	0.49	1.35	0.42	1.49	0.54
Enjoyment	3.06	0.77	2.76	0.65	3.29	0.79
Pride	2.17	0.84	1.85	0.62	2.41	0.91
**Trait constructs**						
Emotional exhaustion	2.02	0.62	2.11	0.61	1.95	0.62
*Emotions–multi-item scales*						
Anger	2.39	0.69	2.48	0.65	2.31	0.72
Anxiety	1.94	0.54	2.08	0.62	1.84	0.45
Shame	1.74	0.44	1.91	0.45	1.60	0.38
Boredom	1.86	0.54	1.84	0.51	1.88	0.57
Enjoyment	3.88	0.52	3.76	0.44	3.97	0.55
Pride	3.51	0.65	3.41	0.61	3.57	0.68
*Emotions–single items (from multi-item scales)*						
Anger	2.52	0.80	2.63	0.81	2.44	0.79
Anxiety	1.30	0.49	1.43	0.57	1.21	0.41
Shame	1.36	0.54	1.53	0.63	1.23	0.43
Boredom	1.67	0.68	1.70	0.70	1.64	0.67
Enjoyment	4.03	0.62	3.90	0.71	4.13	0.62
Pride	3.04	0.83	2.80	0.89	3.23	0.78

*Note*. *n* = 30 female; *n* = 39 male.

**Table 2:** Intercorrelations among Study Measures.

	(1)	(2)	(3)	(4)	(5)	(6)	(7)	(8)	(9)	(10)	(11)	(12)	(13)	(14)
(1) Anger—state	—	.27**	.27**	.24**	-.34**	-.23**								
(2) Anxiety—state	.22	—	.47**	.11**	-.05	.06*								
(3) Shame—state	.26*	.45**	—	.22**	-.03	.09*								
(4) Boredom—state	.32**	.17	.47**	—	-.13**	-.09**								
(5) Enjoyment—state	-.29*	-.13	-.04	-.07	—	.56**								
(6) Pride—state	-.28*	-.03	.13	-.02	.68**	—								
(7) Anger—trait	.42**	.10	.10	.12	-.32**	-.26*	—							
(8) Anxiety—trait	.28*	.20	.12	.10	-.22	-.10	.61**	—						
(9) Shame—trait	.11	.29*	.14	-.02	-.13	-.08	.36**	.53**	—					
(10) Boredom—trait	.27*	.15	.27	.50**	-.16	-.09	.45**	.29*	.16	—				
(11) Enjoyment—trait	-.36**	-.19	-.27*	-.15	.44**	.37**	-.60**	-.48**	-.32**	-.37**	—			
(12) Pride—trait	-.16	-.05	.03	-.01	.26*	.38**	-.36**	-.18	-.13	-.08	.59**	—		
(13) Emot. Exhaustion	.31**	.08	.14	.27*	-.35**	-.22	.58**	.63**	.38**	.50**	-.43**	-.14	—	
(14) Gender	.01	-.03	.02	-.14	-.34**	-.33**	.12	.22	.35**	-.04	-.20	-.12	.13	—

*Note*. State emotions: Single items; trait emotions and emotional exhaustion: Multi-item scales. Gender: 0 = male, 1 = female. For the state emotions: Values above the diagonal show correlations on the within-level (Level 1), values below the diagonal indicate relations on the between level (Level 2). *N*_Level 1_ = 1,089; *N*_Level 2_ = 69.

* *p* < .05.

**p < .01.

**Table 3:** ICC(1) and ICC(2) of the State Emotion Measures.

	ICC(1)	ICC(2)
Anger	.200	.798
Anxiety	.206	.804
Shame	.238	.831
Boredom	.273	.856
Enjoyment	.368	.902
Pride	.435	.924

*Note*. Average number of observations within clusters (measures within persons): 15.78. *N*_Level 1_ = 1,089; *N*_Level 2_ = 69.

#### Teaching Emotions–Trait

Trait teaching anger, anxiety, and enjoyment were assessed using multi-item scales from the Teacher Emotion Scales (TES) [23, 24], and trait teaching shame, boredom, and pride were assessed using adapted multi-item scales from the Achievement Emotions Questionnaire—Mathematics (AEQ-M) [25]. Previous studies indicate a high degree of validity for the trait emotions scales used in this study (see [23–25]). Participants were instructed to answer in terms of how they typically felt while teaching. The answer format was a five-point Likert scale ranging from (1) “*strongly disagree*” to (5) “*strongly agree*.” Four negative and two positive teacher emotions were assessed, namely anger (5 items, α = .87; e.g., “Teaching gives me many reasons to get angry”), anxiety (5 items, α = .82; e.g., “When teaching, I am nervous”), shame (6 items, α = .61; e.g., “When teaching, I am embarrassed”), boredom (7 items, α = .85; e.g., “As I am bored, I find my mind wandering during teaching”), enjoyment (6 items, α = .84; e.g., “I enjoy teaching”), and pride (7 items, α = .86; e.g., “When teaching, I am proud”). Each emotion scale included one item with a similar wording and answer format equivalent to the emotion item in the state assessment allowing for a direct comparison of levels of trait and state emotions (“*How much* [EMOTION] *do you generally experience during teaching?*”). Means and standard deviations for the emotion scales and the particular items serving direct comparison for the state assessment are shown in Table 1, with their intercorrelations outlined in Table 2.

#### Emotional Exhaustion–Trait

Teachers’ emotional exhaustion was assessed via the German version [26] of the respective adapted subscale of the Maslach Burnout Inventory [27]. The scale consisted of nine items (e.g., “*I feel emotionally drained from my work*”) and showed a good reliability (α = .86; mean and standard deviation for the scale are presented in Table 1 and intercorrelations with the other study variables in Table 2). The answer format was a five-point Likert scale ranging from (1) “*strongly disagree*” to (5) “*strongly agree*.”

### Data analysis

To evaluate the two main study hypotheses, a multi-level modeling approach was adopted to account for the nested structure of the data. HLM 6.08 software [28] was used to conduct multi-level analyses comprising two levels (measures nested within teachers). This analytical approach is consistent with those used in previous studies on differences in state versus trait reports of emotional experiences in educational settings [12, 13, 29].

The mixed equation for the model used to examine the discrepancy in levels of state versus trait assessments was as follows:

$${\text{Emotion}}_{\text{ij}}=\gamma_{00}+\gamma_{10}\times {\text{State/Trait}}_{\text{ij}}+u_{0\text{j}}+e_{\text{ij}}$$

Teachers’ emotion scores served as the outcome variable and each included two types of measures within teachers, namely multiple state measures and one trait measure (value of the single-item emotion measure). As the dependent variable contained both state and trait measures, it was important to include in all analyses a variable that differentiated whether the dependent measure score was obtained via a state or trait assessment. Thus, we created the *State/Trait* variable (Level 1, uncentered) that differentiated between the type of measure employed (0 = state, 1 = trait). Most importantly, the coding of this variable allowed its effect (γ_10_) to be interpreted as the discrepancy between state and trait emotion scores, with positive values indicating that trait scores were higher than state scores. For the analyses, it was crucial to use trait and state measures that could be directly compared with respect to mean levels. Thus, all analyses were conducted using trait single items (from the respective trait scales) for which equivalent state single items with identical wordings and answer formats were available. As these parallel item formulations allow for direct comparisons of emotion levels as a function of assessment method, a significant positive effect of the State/Trait variable would indicate that teachers reported higher levels of their trait emotions as compared to their state emotions.

The mixed equation for the model explaining the mean level discrepancies in state versus trait assessments, while controlling for gender, was as follows:

$${\text{Emotion}}_{\text{ij}}=\gamma_{00}+\gamma_{10}\times {\text{State/Trait}}_{\text{ij}}+\gamma_{01}\times {\text{Exhaustion}}_{\text{j}}+\gamma_{02}\times {\text{Gender}}_{\text{j}}+\gamma_{11}\times {\text{State/Trait}}_{\text{ij}}\times {\text{Exhaustion}}_{\text{j}}+\gamma_{12}\times {\text{State/Trait}}_{\text{ij}}\times {\text{Gender}}_{\text{j}}+u_{0\text{j}}+u_{1\text{j}}\times {\text{State/Trait}}_{\text{ij}}+e_{\text{ij}}$$

Similar to the model above, teachers’ emotion scores served as the outcome variable and the *State/Trait* variable (Level 1) differentiated between the type of measure used to assess the outcome variable (0 = *state*, 1 = *trait*). As for the trait emotion scores the means of the multi-item emotion scales were used as those values can be assumed to represent the most reliabile and valid measures of trait emotions in our study (see [30]). The *State/Trait* variable differentiated between the method of assessment (0 = *state*, 1 = *trait*; uncentered) with this variable’s effect (γ_10_) being interpretable as the discrepancy between state and trait emotion scores, with positive values indicating that trait scores were higher than state scores. As per the coding of the State/Trait variable (0 = state, 1 = trait), the γ_00_ intercept represents the overall mean state emotional experience when the values for other linear terms (Level 2 variables included in the H2 analyses) also are zero.

Two *Level 2 variables* were included in our models, namely *Exhaustion* (γ_01_, *z*-standardized across teachers) and *Gender* (control variable; 0 = *male*, 1 = *female*; γ_02_, uncentered). Finally, two *cross-level multiplicative interaction terms* were included in our models, namely *State/Trait* × *Exhaustion* (γ_11_) and *State/Trait* × *Gender* (γ_12_). These interaction terms represent the effects of Exhaustion and Gender (control variable) on the discrepancy in state versus trait emotion scores.

The model for each emotion was calculated as a “slopes-as-outcome model” [31]. It examined the effect of the State/Trait × Exhaustion interaction (γ_11_) as well as the effect of the State/Trait × Gender interaction (γ_12_) as predictors of the discrepancy between state and trait emotion scores (i.e., the slope). In the model, the corresponding main effects were also included (γ_01_, γ_02_). Accordingly, the way in which our models were constructed allowed us to infer the extent to which the discrepancy between state and trait emotions could be explained by emotional exhaustion while controlling for gender.

## Results

### Discrepancy in Levels of State versus Trait Assessments

The results for the analysis on the discrepancy in levels of state versus trait assessments are outlined in Table 4. The main effect of assessment method (State/Trait variable; γ_10_) on the emotion scores was positive and highly significant for each of the six emotions. The magnitude of the positive effects observed indicates for each emotion how much higher the trait score is as compared to the state score (Intercept γ_00_). It is important to note that the two assessment methods (state, trait) can directly be compared as they are based on parallel item wordings and identical answer formats. The findings thus support our hypothesis in showing teachers to report higher levels of trait emotions as compared to state emotional experiences. Concerning the relative magnitude of the intensity levels, our findings are also in line with previous studies showing both state (γ_00_) and trait levels (γ_00_ + γ_10_) for the positive emotions to be higher than for the negative emotions, with enjoyment and anger representing the strongest positive and negative teaching emotions, respectively.

**Table 4:** Differences between Teachers’ State and Trait Reports of Emotions.

Level and predictor	Anger	Anxiety	Shame	Boredom	Enjoyment	Pride
**Level 1**						
Intercept (γ_00_)	1.61***	1.08***	1.09***	1.44***	3.05***	2.16***
	(0.06)	(0.02)	(0.02)	(0.06)	(0.09)	(0.10)
State/Trait (γ_10_)	0.91***	0.22**	0.28***	0.23**	0.98***	0.88***
	(0.10)	(0.06)	(0.06)	(0.09)	(0.09)	(0.11)
**Variance components**						
Within-student (L1) variance (ơ^2^)	0.729	0.089	0.095	0.517	0.801	0.782
Intercept (L2) variance (τ_00_)	0.188	0.023	0.030	0.197	0.489	0.617
Slope (L2) variance (τ_11_)	0.031	0.160	0.147	0.027	0.193	0.162
Intercept-slope (L2) covariance (τ_01_)	-0.071	-0.014	0.011	-0.070	-0.305	-0.313

*Note*. Values in brackets: Standard errors. State/Trait: 0 = state, 1 = trait; *N*_Level 1_ = 1,158 (resulting from 1,089 state assessments and 69 trait assessments); *N*_Level 2_ = 69.

** *p* < .01.

*** *p* < .001.

### Explaining Mean Level Discrepancies in State versus Trait Assessments

The results of the analyses to explain mean level discrepancies in state versus trait assessments are outlined in Table 5. Similar to the results for the first hypothesis, the main effect of assessment method (State/Trait variable; γ_10_) on the emotion scores was positive and highly significant for all six emotions showing all trait scores based on multi-item scales to be higher than the reported state scores (Intercept γ_10_). Most important with respect to our hypothesis, the effects of the State/Trait × Exhaustion interaction (γ_11_) were positive and significant for each of the four negative emotions showing greater emotional exhaustion to predict higher discrepancies between the trait and the state self-reports of negative emotions. The State/Trait × Exhaustion interaction (γ_11_) was not significant for positive emotions, showing emotional exhaustion levels to not explain differences between state and trait self-reported positive emotion. Concerning the moderating effect of gender (control variable), the State/Trait × Gender interaction (γ_12_) reached significance for shame, showing higher levels of trait versus state self-reported shame to be more pronounced for female teachers. Results of the analyses without gender as a control variable were nearly identical to those with gender included as a covariate and supported our hypothesis (see S1 Table).

**Table 5:** Predicting Teachers’ Emotions: Results from Multilevel Modeling.

	Anger	Anxiety	Shame	Boredom	Enjoyment	Pride
**Level 1**						
Intercept (γ_00_)	1.62***	1.09***	1.09***	1.51***	3.25***	2.39***
	(0.08)	(0.03)	(0.03)	(0.09)	(0.11)	(0.13)
State/Trait (γ_10_)	0.73***	0.79***	0.53***	0.39***	0.70***	1.17***
	(0.10)	(0.06)	(0.06)	(0.09)	(0.10)	(0.13)
**Level 2**						
Exhaustion (γ_01_)	0.16**	0.02	0.03	0.14	-0.26**	-0.16
	(0.05)	(0.02)	(0.02)	(0.08)	(0.07)	(0.09)
Gender (γ_02_)	-0.05	-0.01	0.00	-0.17	-0.45**	-0.51**
	(0.11)	(0.04)	(0.05)	(0.12)	(0.15)	(0.18)
**Cross-level interactions L1-L2**						
State/Trait × Exhaustion (γ_11_)	0.24***	0.31***	0.13**	0.14*	0.06	0.09
	(0.06)	(0.05)	(0.05)	(0.05)	(0.06)	(0.10)
State/Trait × Gender (γ_12_)	0.11	0.16	0.27*	0.05	0.30	0.36
	(0.15)	(0.10)	(0.10)	(0.11)	(0.16)	(0.19)
**Variance components**						
Within-student (L1) variance (ơ^2^)	0.713	0.089	0.095	0.505	0.798	0.772
Intercept (L2) variance (τ_00_)	0.172	0.024	0.030	0.181	0.353	0.530
Slope (L2) variance (τ_11_)	0.066	0.089	0.095	0.048	0.189	0.246
Intercept-slope (L2) covariance (τ_01_)	-0.105	-0.007	-0.019	-0.092	-0.257	-0.360

*Note*. Values in brackets: Standard errors. State/Trait: 0 = state, 1 = trait; Gender: 0 = male, 1 = female; *N*_Level 1_ = 1,158 (1,089 state assessments, 69 trait assessments); *N*_Level 2_ = 69.

* *p* < .05.

** *p* < .01.

*** *p* < .001.

In addition to results specific to explaining the mean level discrepancies in state versus trait assessments, the main effect of emotional exhaustion on the emotion scores (γ_01_) was significant for anger (positive effect) and enjoyment (negative effect) showing teachers who reported greater exhaustion to also report higher levels of anger and lower levels of enjoyment. Further, the main effect of Gender (control variable; γ_02_) was significant for enjoyment and pride (negative effects) showing female teachers to report lower levels of pride and enjoyment relative to males. In sum, the results observed when evaluating self-reported exhaustion as a moderating variable provided support for hypothesis in showing emotional exhaustion to explain a substantial amount of the state-trait discrepancy in self-reports of negative emotions in teachers.

## Discussion

To our knowledge, this study is the first to examine the discrepancy in teachers’ state (momentary) versus trait (habitual) teaching emotions, as well as the potential moderating effects of emotion-related beliefs on the state-trait discrepancies. In line with previous results on differences in trait versus state levels of students’ emotions (e.g., [12]), intensity or impact bias (see [16, 32]), and initial findings on trait and state levels in teaching emotions [10], we found teachers to report higher levels of their negative and positive trait emotions in the classroom as compared to their self-reported state emotions (see [4]). Consistent with the accessibility model of emotion self-report [6], individual differences in the state-trait discrepancy in negative emotions could be explained by teachers’ perceptions of emotional exhaustion. Specifically, we found emotional exhaustion to positively predict the discrepancy between trait versus state levels of reported anger, anxiety, shame, and boredom. In contrast, no moderating effect of emotional exhaustion on the state-trait discrepancy in enjoyment and pride was observed.

### Teachers’ State-trait Discrepancy in Reports of Teaching Emotions

Our results consistently showed teachers to report higher levels of both their negative and positive trait teaching emotions as compared to their self-reported emotions on the real-time state measures. This discrepancy was also notably strong, with effect size estimates (Cohen’s *d*) [33] ranging from *d* = 0.40 (boredom) to *d* = 1.39 (enjoyment; mean *d* = 0.91).

With respect to the higher levels in reports of trait emotions, it is important to acknowledge that although trait measures may not be as ecologically valid as real-time self-reports, individuals’ beliefs concerning the intensity of their typical emotional experiences as reported on trait measures are nonetheless important. For example, a study by Wirtz, Kruger, Napa Scollon, and Diener [34] found trait reports to be even more predictive of decision-making than state reports. As such, it can reasonably be assumed that teachers’ decisions in the classroom context, for example, with respect to their instructional strategies, professional development, or quitting intentions, may more strongly depend on their overarching beliefs concerning their teaching emotions than on their actual emotional experiences in the classroom [4]. Additionally, our findings are important for researchers in providing valuable insight into the underlying reasons for possible discrepancies between studies employing trait measures of teachers’ emotions or qualitative interviews (e.g., [35–38]) and those utilizing real-time self-reports or objective indicators (e.g., behavioral or physiological measures)

### Exhaustion as a Predictor of the State-trait Discrepancy in Negative Teaching Emotions

In line with the second study hypothesis, teachers’ reports of their emotional exhaustion proved to be a salient identity-related belief (i.e., a belief about one’s emotions in general) within teachers’ negative belief system that significantly moderated the state-trait discrepancy for negative emotions: The higher teachers’ emotional exhaustion levels, the stronger their state-trait discrepancy in reports of anger, anxiety, shame, and boredom. Thus, the present study findings contribute not only to our understanding of the extent to which trait emotion measures may not reliably account for teachers’ intensity of their real-time emotions in the classroom, but also the degree to which higher levels of reported negative emotions may be based on teachers’ exhaustion levels.

As it is unclear in the research literature whether emotional exhaustion represents a salient identity-related belief with respect to individuals’ positive emotions, no hypotheses were proposed with respect to exhaustion moderating the state-trait discrepancy in enjoyment and pride. No moderation effect was found for these emotions in our study. Interpreted in the framework of Robinson and Clore’s [6] accessibility model, emotional exhaustion therefore does not appear to be a salient belief with respect to reports of positive trait emotions, suggesting that this belief instead may operate primarily within teachers’ negative belief system. In contrast, the effect of emotional exhaustion on teachers’ reports of trait emotions was rather detrimental, particularly given the various adverse cognitive and behavioral consequences of negative emotions (e.g., quitting intentions) (see [34]) and greater impact of negative emotions relative to positive emotions on a range of psychological phenomena (e.g., social network patterns, learning processes) (see [39]).

### Reciprocal Effects of Emotional Exhaustion and Negative Trait Emotions

Although our study is not longitudinal or experimental in nature, and consequently does not allow for causal relations to be examined, it can be assumed that teachers’ judgments of their emotional exhaustion are likely predictors of the state-trait discrepancy in their reports of negative teaching emotions. Such emotions, in turn, represent an important source of information (above and beyond other factors such as sleep quality, ability to concentrate, etc.) with respect to subsequent judgments of emotional exhaustion. As such, it is possible that a feedback loop between one’s emotional exhaustion and beliefs about negative emotions (i.e., negative trait emotions; both belonging to the negative belief system) could result in a downward spiral and result in increasingly problematic levels of both exhaustion and negative trait teaching emotions over time. This feedback loop might additionally contribute to the various detrimental effects of negative emotions and burnout in teachers such as physical health problems (e.g., [40]) or high dropout rates (see [41]).

With respect to ways in which such a downward spiral may be minimized or prevented, it is possible that encouraging teachers to accurately reflect on their real-time emotions in the classroom. For example, teacher reflection could be facilitated through ESM approaches similar to those employed in this study as well as post-class emotion diaries (less intrusive during instruction) or reviewing objective physiological records (e.g., heart rate bracelet). Similar to bio-feedback techniques in cognitive behavioral therapy [42], providing teachers more proximal and reliable information concerning their negative state emotions while teaching could help to reduce the state-trait discrepancy in reports of negative emotions that is otherwise likely to be subject to recall bias (e.g., peak events, primacy/recency effects) and emotion-related beliefs. Whereas it is possible that trait reports of positive emotions might also be reduced given more accurate real-time data, the net effect is assumed to be positive given prior research showing stronger effects of negative relative to positive emotions over time [39].

## Limitations and Conclusion

Concerning the study limitations, it is first important to note that although we examined a large number of assessments within teachers (*N* = 1,089), the Level 2 sample size was restricted to 69 teachers. According to Maas and Hox [43], this is sufficient for our method of analysis, yet future studies should replicate and expand the present findings with a larger and more representative sample at the person level. Second, the present study focused only on emotional exhaustion as an identity-related belief within teachers’ negative belief system due to its prevalence in this population as a critical component of teacher burnout. However, other emotion-related beliefs also warrant investigation in this regard to further elucidate the specific cognitive processes responsible for teachers’ state-trait discrepancy in their reports of the emotions they experience in class. More specifically, further investigation of teachers’ beliefs within their positive belief system (e.g., teacher self-efficacy, personal goals) is important to more fully assess whether competence or value-related beliefs might represent salient moderators of the state-trait discrepancy in teachers emotions, particularly with respect to their positive emotions.

Third, our study was restricted to the assessment of six discrete emotions and focused mainly on the most intensely experienced positive and negative emotions reported by teachers [3, 11]. Therefore, future studies to investigate potential state-trait discrepancies in additional discrete emotions such as hopelessness, guilt, hope, relief, and relaxation in teachers are encouraged to provide a more comprehensive perspective on how teachers’ emotion-related beliefs impact their accounts of other facets of their emotional lives (for such emotion scales which might be adapted for teachers see [25]).

Finally, our sample of 69 teachers was recruited across 27 different subjects, resulting in a relatively low number of assessments related to a specific subject. Therefore, it was not possible to analyze our data in a subject-specific manner. Although the relations as hypothesized in our study are assumed to be rather stable across subjects, future studies that compare specific subject domains (e.g., mathematics vs. a language domain) to investigate domain-related differences in mean levels and structural relations are recommended.

In sum, the present study findings contribute to our understanding of how the intensity of teachers’ emotions may differ based on state versus trait assessments, how teachers’ reports of trait emotions may not accurately reflect their lived emotional experiences in the classroom, and further, how teachers’ emotional exhaustion may represent an important set of emotion-related beliefs that account for individual differences in the state-trait discrepancy in reports of negative teaching emotions. Given the potential for a deleterious downward spiral of mutually enhancing emotional exhaustion and reports of negative trait emotions over time, research on the potential benefits of state-emotion feedback (e.g., bio-feedback) is recommended in an effort to reduce dropout rates as well as improve the emotional lives of teachers.

## Supporting Information

S1 File Data Set. This zipped folder contains two SPSS files with the original datasets of the study (S1_L1.sav for state data and S1_L2.sav for trait data).
(ZIP)

S1 Table Additional Analyses. This word document contains additional analyses (Results from Multilevel Modeling Predicting Teachers’ Emotions with No Covariate Included).
(DOCX)
