# Supplementary material for: The Glass Half Empty: How Emotional Exhaustion Affects the State-Trait Discrepancy in Self-Reports of Teaching Emotions
Source: PLoS One. 2015 Sep 14;10(9):e0137441. doi: 10.1371/journal.pone.0137441 (PMC4569532; doi:10.1371/journal.pone.0137441)
Supplement: S1 Table — This word document contains additional analyses (Results from Multilevel Modeling Predicting Teachers’ Emotions with No Covariate Included). (DOCX) [file pone.0137441.s002.docx]

S1 Table

Additional Analyses

Table S1. Predicting Teachers’ Emotions: Results from Multilevel Modeling

|  | Anger | Anxiety | Shame | Boredom | Enjoyment | Pride |
| --- | --- | --- | --- | --- | --- | --- |
| **Level 1** |  |  |  |  |  |  |
| Intercept (γ_00_) | 1.60*** | 1.08*** | 1.09*** | 1.44*** | 3.05*** | 2.17*** |
|  | (0.06) | (0.02) | (0.02) | (0.06) | (0.08) | (0.10) |
| State/Trait (γ_10_) | 0.78*** | 0.86*** | 0.65*** | 0.42*** | 0.83*** | 1.33*** |
|  | (0.07) | (0.05) | (0.05) | (0.06) | (0.08) | (0.10) |
| **Level 2** |  |  |  |  |  |  |
| Exhaustion (γ_01_) | 0.15** | 0.02 | 0.03 | 0.13 | -0.29*** | -0.19* |
|  | (0.05) | (0.02) | (0.02) | (0.07) | (0.07) | (0.09) |
| **Cross-level interactions L1-L2** |  |  |  |  |  |  |
| Trait/State × Exhaustion (γ_11_) | 0.25*** | 0.32*** | 0.14** | 0.14* | 0.07 | 0.11 |
|  | (0.06) | (0.06) | (0.05) | (0.05) | (0.07) | (0.11) |
| **Variance components** |  |  |  |  |  |  |
| Within-student (L1) variance (ơ^2^) | 0.713 | 0.089 | 0.095 | 0.505 | 0.797 | 0.772 |
| Intercept (L2) variance (τ_00_) | 0.169 | 0.023 | 0.029 | 0.184 | 0.398 | 0.585 |
| Slope (L2) variance (τ_11_) | 0.064 | 0.078 | 0.084 | 0.047 | 0.205 | 0.268 |
| Intercept-slope (L2) covariance (τ_01_) | -0.105 | -0.004 | -0.025 | -0.092 | -0.284 | -0.394 |

*Note*. Values in brackets: Standard errors. State/Trait: 0 = state, 1 = trait; *N*_Level 1_ = 1,158 (1.089 state assessments, 69 trait assessments); *N*_Level 2_ = 69.

* *p* < .05. ** *p* < .01. *** *p* < .001.
